# Supplementary material for: On the mechanistic nature of epistasis in a canonical cis-regulatory element
Source: eLife. 2017 May 18;6:e25192. doi: 10.7554/eLife.25192 (PMC5481185; doi:10.7554/eLife.25192)
Supplement: Supplementary file 1. — DOI: http://dx.doi.org/10.7554/eLife.25192.016 [file elife-25192-supp1.docx]

**Supplementary File 1. Identity of randomly generated double mutants**, ordered by mutated residue number.

| **Mutated residue number**  **wildtype identity:** | 1 10 20 30 40  -35 -10___  TAACACCGTGCGTGTTGACTATTTTACCTCTGGCGGTGATAAT  *O_R2_  O_R1_* | | | |
| --- | --- | --- | --- | --- |
| **Double mutant** | **Mutated residue #1** | **New residue #1 identity** | **Mutated residue #2** | **New residue #2 identity** |
| 1 | 1 | C | 3 | T |
| 2 | 1 | A | 7 | T |
| 3 | 1 | A | 10 | A |
| 4 | 1 | A | 11 | A |
| 5 | 1 | C | 11 | T |
| 6 | 1 | A | 11 | T |
| 7 | 1 | C | 12 | A |
| 8 | 1 | A | 15 | C |
| 9 | 1 | A | 19 | T |
| 10 | 1 | A | 24 | A |
| 11 | 1 | A | 34 | T |
| 12 | 1 | A | 37 | C |
| 13 | 1 | C | 40 | C |
| 14 | 1 | A | 40 | A |
| 15 | 1 | A | 43 | A |
| 16 | 2 | G | 4 | T |
| 17 | 2 | G | 22 | C |
| 18 | 2 | G | 36 | A |
| 19 | 2 | G | 42 | T |
| 20 | 3 | T | 5 | G |
| 21 | 3 | T | 7 | A |
| 22 | 3 | C | 16 | A |
| 23 | 3 | G | 34 | T |
| 24 | 3 | T | 36 | A |
| 25 | 3 | G | 42 | T |
| 26 | 4 | T | 11 | T |
| 27 | 4 | T | 13 | C |
| 28 | 4 | T | 15 | C |
| 29 | 4 | T | 17 | T |
| 30 | 4 | G | 18 | T |
| 31 | 4 | T | 24 | C |
| 32 | 5 | G | 14 | A |
| 33 | 5 | T | 21 | T |
| 34 | 5 | G | 34 | T |
| 35 | 5 | G | 34 | A |
| 36 | 5 | G | 41 | G |
| 37 | 6 | G | 15 | C |
| 38 | 6 | T | 18 | T |
| 39 | 6 | T | 22 | C |
| 40 | 6 | T | 24 | A |
| 41 | 6 | T | 28 | G |
| 42 | 6 | T | 39 | T |
| 43 | 6 | G | 39 | G |
| 44 | 7 | T | 10 | A |
| 45 | 7 | A | 13 | C |
| 46 | 7 | T | 14 | A |
| 47 | 7 | A | 17 | A |
| 48 | 7 | A | 20 | C |
| 49 | 7 | T | 21 | G |
| 50 | 7 | A | 24 | A |
| 51 | 8 | A | 12 | A |
| 52 | 8 | A | 15 | A |
| 53 | 8 | A | 16 | C |
| 54 | 8 | A | 20 | G |
| 55 | 8 | A | 29 | A |
| 56 | 8 | A | 36 | A |
| 57 | 8 | A | 39 | G |
| 58 | 9 | C | 13 | A |
| 59 | 9 | C | 16 | A |
| 60 | 9 | A | 21 | G |
| 61 | 9 | C | 23 | A |
| 62 | 9 | A | 23 | C |
| 63 | 9 | A | 24 | C |
| 64 | 9 | A | 35 | T |
| 65 | 9 | A | 35 | T |
| 66 | 9 | A | 41 | T |
| 67 | 10 | A | 17 | A |
| 68 | 10 | A | 24 | C |
| 69 | 11 | T | 16 | C |
| 70 | 11 | T | 38 | A |
| 71 | 11 | T | 39 | T |
| 72 | 12 | A | 16 | A |
| 73 | 12 | A | 18 | T |
| 74 | 12 | A | 39 | T |
| 75 | 13 | A | 18 | G |
| 76 | 13 | C | 19 | G |
| 77 | 13 | C | 19 | T |
| 78 | 13 | C | 23 | A |
| 79 | 13 | C | 27 | T |
| 80 | 13 | C | 43 | A |
| 81 | 15 | A | 20 | A |
| 82 | 15 | A | 28 | G |
| 83 | 15 | A | 29 | A |
| 84 | 15 | C | 29 | A |
| 85 | 15 | A | 31 | C |
| 86 | 15 | A | 40 | C |
| 87 | 16 | C | 18 | T |
| 88 | 16 | G | 21 | G |
| 89 | 16 | A | 22 | A |
| 90 | 16 | C | 23 | C |
| 91 | 16 | A | 23 | A |
| 92 | 16 | C | 24 | A |
| 93 | 16 | C | 36 | A |
| 94 | 16 | C | 40 | C |
| 95 | 16 | C | 41 | G |
| 96 | 17 | T | 20 | C |
| 97 | 17 | A | 23 | C |
| 98 | 17 | A | 34 | T |
| 99 | 17 | T | 39 | G |
| 100 | 18 | T | 19 | G |
| 101 | 18 | T | 24 | A |
| 102 | 18 | G | 34 | A |
| 103 | 18 | T | 34 | T |
| 104 | 18 | G | 34 | T |
| 105 | 18 | T | 39 | T |
| 106 | 18 | G | 41 | G |
| 107 | 19 | G | 34 | A |
| 108 | 19 | T | 35 | A |
| 109 | 19 | T | 37 | C |
| 110 | 19 | T | 41 | C |
| 111 | 20 | G | 24 | A |
| 112 | 20 | A | 26 | G |
| 113 | 20 | A | 42 | G |
| 114 | 20 | A | 42 | G |
| 115 | 22 | A | 23 | A |
| 116 | 22 | C | 23 | C |
| 117 | 22 | A | 26 | T |
| 118 | 22 | A | 37 | A |
| 119 | 22 | C | 37 | A |
| 120 | 22 | A | 42 | G |
| 121 | 23 | C | 24 | A |
| 122 | 23 | C | 30 | T |
| 123 | 23 | C | 34 | T |
| 124 | 23 | A | 34 | T |
| 125 | 23 | C | 35 | A |
| 126 | 23 | A | 41 | T |
| 127 | 24 | C | 25 | C |
| 128 | 24 | C | 34 | T |
| 129 | 24 | A | 34 | T |
| 130 | 25 | A | 29 | A |
| 131 | 25 | A | 41 | G |
| 132 | 26 | T | 40 | C |
| 133 | 29 | C | 37 | A |
| 134 | 29 | A | 42 | G |
| 135 | 31 | C | 41 | G |
| 136 | 31 | C | 42 | T |
| 137 | 32 | T | 41 | G |
| 138 | 33 | T | 38 | A |
| 139 | 37 | C | 41 | T |
| 140 | 41 | T | 42 | T |
| 141 | 42 | T | 43 | A |
