# Supplementary material for: On the mechanistic nature of epistasis in a canonical cis-regulatory element
Source: eLife. 2017 May 18;6:e25192. doi: 10.7554/eLife.25192 (PMC5481185; doi:10.7554/eLife.25192)
Supplement: Supplementary file 2. — DOI: http://dx.doi.org/10.7554/eLife.25192.017 [file elife-25192-supp2.docx]

**Supplementary File 4. Types of epistasis in two environments.** In the absence of CI repressor, magnitude epistasis - the sign of single mutation effects and of the double mutant is the same - predominates. Sign epistasis (sign of the double mutant is different to the sign of one of the single mutants), and reciprocal sign epistasis (sign of the double mutant is different to both single mutant effects) are rare. In the presence of CI repressor, sign and reciprocal sign epistasis are common. Only the double mutants with significant epistasis are counted.

|  | **Magnitude epistasis** | **Reciprocal sign epistasis** | **Sign epistasis** |
| --- | --- | --- | --- |
| **Absence of CI** | 94 | 9 | 10 |
| **Presence of CI** | 4 | 39 | 16 |
