# Supplementary material for: On the mechanistic nature of epistasis in a canonical cis-regulatory element
Source: eLife. 2017 May 18;6:e25192. doi: 10.7554/eLife.25192 (PMC5481185; doi:10.7554/eLife.25192)
Supplement: Supplementary file 3. — DOI: http://dx.doi.org/10.7554/eLife.25192.018 [file elife-25192-supp3.docx]

**Supplementary File 5. Identity of mutants used for verification of the model,** ordered by residue number.

| **Mutated residue number**  **wildtype identity:** | 1 10 20 30 40  -35 -10___  TAACACCGTGCGTGTTGACTATTTTACCTCTGGCGGTGATAAT  *O_R2_  O_R1_* |
| --- | --- |

| **Double mutant**  **(mv – model verification)** | **Sign of individual mutation effects (as per Table 1)** | **Mutated residue #1** | **Mutant residue #1 identity** | **Mutated residue #2** | **Mutant residue #2 identity** |
| --- | --- | --- | --- | --- | --- |
| mv1 | - + \| + - | 17 | A | 27 | G |
| mv2 | - + \| + - | 34 | A | 39 | G |
| mv3 | - + \| + - | 27 | G | 39 | T |
| mv4 | - + \| + - | 17 | A | 33 | T |
| mv5 | - + \| + - | 33 | T | 39 | G |
| mv6 | + - \| + - | 30 | T | 38 | A |
| mv7 | + - \| + - | 30 | T | 34 | A |
| mv8 | + - \| + - | 27 | G | 34 | A |
| mv9 | + - \| + - | 27 | G | 33 | T |
| mv10 | + - \| + - | 33 | T | 38 | A |
| mv11 | - + \| - + | 17 | A | 39 | G |
| mv12 | - + \| - + | 3 | C | 15 | G |
| mv13 | - + \| - + | 10 | C | 17 | A |
| mv14 | - + \| - + | 11 | T | 39 | T |
| mv15 | - + \| - + | 11 | G | 39 | G |
| mv16 | - - \| - - | 16 | C | 36 | A |
| mv17 | - - \| - - | 15 | A | 31 | C |
| mv18 | - - \| - - | 25 | A | 41 | G |
| mv19 | - - \| - - | 26 | T | 40 | C |
| mv20 | - - \| - - | 16 | C | 41 | G |
| mv21 | - - \| - + | 9 | C | 16 | A |
| mv22 | - - \| - + | 25 | A | 29 | A |
| mv23 | - - \| - + | 15 | A | 29 | A |
| mv24 | - - \| - + | 15 | C | 29 | A |
| mv25 | - - \| - + | 17 | A | 34 | T |
| mv26 | - - \| + - | 32 | T | 41 | G |
| mv27 | - - \| + - | 15 | A | 34 | A |
| mv28 | - - \| + - | 25 | C | 33 | T |
| mv29 | - - \| + - | 37 | A | 38 | A |
| mv30 | - - \| + - | 32 | T | 40 | C |
